# Circulating microRNAs and hepcidin as predictors of iron homeostasis and anemia among school children: a biochemical and cross-sectional survey analysis

**DOI:** 10.1186/s40001-023-01579-5

**Published:** 2023-12-15

**Authors:** Hadeel A. Al-Rawaf, Sami A. Gabr, Amir Iqbal, Ahmad H. Alghadir

**Affiliations:** 1 Departments of Clinical Laboratory Sciences, College of Applied Medical Sciences, King Saud University, Riyadh, Saudi Arabia; 2 Rehabilitation Research Chair, Department of Rehabilitation Sciences, College of Applied Medical Sciences, King Saud University, Riyadh, Saudi Arabia

**Keywords:** Acute phase reaction, Subclinical inflammation, Iron status, School children, Adolescence, Micro-RNAs

## Abstract

**Background:**

MicroRNAs (miRNAs) can control several biological processes. Thus, the existence of these molecules plays a significant role in regulating human iron metabolism or homeostasis.

**Purpose:**

The study aimed to determine the role of circulating microRNAs and hepcidin in controlling iron homeostasis and evaluating possible anemia among school children.

**Methods:**

The study was based on a biochemical and cross-sectional survey study that included three hundred fifty school children aged 12–18 years old. RT–PCR and immunoassay analysis were accomplished to estimate iron concentration, Hgb, serum ferritin (SF), soluble transferrin receptor (sTfR), total body iron stores (TIBs), total oxidative stress (TOS), total antioxidant capacity (TAC), α-1-acid glycoprotein (AGP), high sensitive C-reactive protein (hs-CRP), and miRNAs; miR-146a, miR-129b, and miR-122 in 350 school adolescents.

**Results:**

Iron disorders were cross-sectionally predicted in 28.54% of the study population; they were classified into 14.26% with ID, 5.7% with IDA, and 8.6% with iron overload. The overall proportion of iron depletion was significantly higher in girls (20.0%) than in boys (8.6%). MicroRNAs; miR-146a, miR-125b, and miR-122 were significantly upregulated with lower hepcidin expression in adolescence with ID and IDA compared to iron-overloaded subjects, whereas downregulation of these miRNAs was linked with higher hepcidin. Also, a significant correlation was recorded between miRNAs, hepcidin levels, AGP, hs-CRP, TAC, and other iron-related indicators.

**Conclusion:**

Molecular microRNAs such as miR-146a, miR-125b, and miR-122 were shown to provide an additional means of controlling or regulating cellular iron uptake or metabolism either via the oxidative stress pathway or regulation of hepcidin expression via activating genes encoding Hfe and Hjv activators, which promote iron regulation. Thus, circulating miRNAs as molecular markers and serum hepcidin could provide an additional means of controlling or regulating cellular iron and be associated as valuable markers in diagnosing and treating cases with different iron deficiencies.

## Introduction

In Childhood, anaemia is considered one of the most widespread health problems, significantly associated with the incidence of iron deficiency (ID) as a significant nutritional problem worldwide [1–3]. In developing and non-developing countries, in various African and Asian settings, a higher prevalence of anaemia (around 40%) in contribution to iron deficiency was reported among children [4].

In particular, iron deficiency anaemia leads to severe health problems such as general weakness in body growth, a compromise in the immune system’s ability, increased susceptibility to chronic infections, and increased morbidity. Also, it causes poor cognitive performance and delays in psychomotor development, resulting in lower schooling achievements [5, 6]. In most clinical practices, the most commonly utilized markers of iron deficiency were haemoglobin (Hgb), iron concentration, serum ferritin (SF), and soluble transferrin receptor (sTfR), the levels of which efficiently reflect intracellular iron stores [2, 7, 8]. Previous studies showed that SF and sTfR underwent a characteristic sequence of changes following a decrease in body iron stores from typical iron values to those found in ID [9–12].

Past studies reported that “an interference with micronutrient status, especially biomarkers of iron status were previously reported among healthy populations, especially those of acute phase reaction or subclinical inflammation” [13–15]. Thus, “three phases of subclinical infection and inflammation like incubation, early, and late convalescence phase were identified by specialized monitoring markers such as C-reactive protein (CRP) and α-1-acid glycoprotein (AGP), respectively” [16]. In particular, “it was suggested previously that the concentrations of most iron indicators such as serum ferritin can be easily affected by inflammation caused by the acute-phase response (APR), infection, injury, or environmental insults” [17]. Little is known about cellular biological mechanisms explaining the influence of inflammation on the expression of the biological indicators of iron status [17, 18]. “It was reported that regulation, control, and synthesis of hepatic acute phase proteins (APS), such as ferritin, transferrin, haptoglobin, and hepcidin, were shown to proceed via inflammatory pathways. It can be induced by the acute-phase response (APR) and may affect the distribution of iron to cells throughout the body” [17–19]. Recently, both inflammation and different phases of the APR may lead to an overestimation of Fe status and significantly initiate opposite effects on assessing Fe status using TfR [20].

In most cases with iron disorders, especially in cases with overweight and obesity, ferritin was considered as a marker of inflammation rather than iron status and other related markers; transferrin, iron concentrations, sTfR, and Hgb alone can truly predict iron deficiency in such people [21]. As a newly discovered harmful iron regulatory hormone, hepcidin can explain iron deficiency and anaemia, particularly in cases with chronic inflammation and infections [22].

Hepcidin from the liver cells was expressed and released from liver cells after stimulation with proinflammatory cytokines such as interleukin-6. Liver hepcidin is reported to play a role in iron status by suppressing intestinal iron absorption, releasing storage iron, and controlling the amount of iron available in the circulation [23–26]. This process is accomplished via interacting with the iron exporter ferroportin [23–26]. Thus, during interventions of iron supplementation, the regulation of host iron absorption reflects the master efficiency role of hepcidin in the complete incorporation of iron into erythrocytes [27]. In this regard, different significant iron disorders such as iron deficiency anaemia [28], iron overload disorders [29], or thalassemias [30] frequently arise from the disturbance in the hepcidin/ferroportin regulatory system, which ultimately leads to an imbalance of systemic iron levels.

In vitro studies evaluated the role of hepcidin in iron metabolism. However, human studies showed insufficient data, especially among healthy subjects [31]. Thus, more research studies try to investigate the role of miRNAs in iron homeostasis and possible cases of anaemia in healthy subjects [31]. An abundant class of noncoding short RNAs termed microRNAs (miRNAs) showed significantly to regulate gene expression [32, 33]. In addition, a wide range of biological processes, such as development, differentiation, metabolism, angiogenesis, and vascular remodeling of injured tissues in diabetic and non-diabetic wound healing, was significantly controlled by a set of cellular microRNAs [32, 33].

Computational analyses suggest that up to 30% of the human protein-coding genes were shown to be regulated by miRNAs [34]; however, so far, only a small number of target genes have been experimentally confirmed [35].

Previous studies suggested that miRNA may also have a regulatory effect on human iron homeostasis. The role of miRNA in modulating iron homeostasis is proposed to be at least a part of a heme-dependent process [36, 37]. At multiple points, both systemic and cellular iron homeostasis were potentially regulated by miRNAs by influencing iron absorption, transport, storage, and utilization [38].

Thus, the current study aimed to determine the role of circulating microRNAs and hepcidin in controlling iron homeostasis and evaluating possible anaemia based upon correlation with acute-phase reaction or subclinical inflammation among crossectional survey analysis on schoolchildren aged 12–18.

## Materials and methods

### Subjects

A total of 350 schoolchildren aged 12–18 years, most of them are boys (*n* = 200) and girls (*n* = 150), respectively.“During September 2015 and May 2016, the participants were randomly invited from different Prep public and high schools to participate in this descriptive survey analysis”[39]. Participants “with genetic, endocrine, and cardiovascular disorders or chronic diseases like diabetes, cardiac, pulmonary, and neurological diseases or acute infections were excluded from this study. In addition, participants who received diets or medical therapy that had affected the iron status data were excluded from this study” [39]. After the assignment of written informed consent from the parents of all participating school children, the data and blood samples were collected and kept frozen at – 80 °C until use [39]. Based on the estimation of iron status, our participants were classified into four groups: Normal group (*n* = 250; 75–175 μg/dl), iron deficiency (ID) group (*n* = 50; < 75 μg/dl), iron deficiency with anaemia (IDA) group (*n* = 20; < 75 μg/dl and Hgb < 12.0), and iron overload group (n = 30; high iron levels ˃175 μg/dl). All clinical and demographic data are included in Table 1. The protocol of the current study was reviewed according to the ethical guidelines of the 1975 Declaration of Helsinki and approved by the Ethics Sub-Committee of King Saud University, Kingdom of Saudi Arabia, under file number ID: RRC-2015-072.

**Table 1:** Baseline of clinical and laboratory characteristics for adolescents based on iron scores (n = 350; mean ± SD)

Variables	Iron status				*P*-value
	N I group (*n* = 250)	ID group (*n* = 50)^a^	IDA group (*n* = 20)^b^	IO group (*n* = 30)^c^	
Age	14.6 ± 1.5	14.2 ± 1.7	14.1 ± 1.5	14.3 ± 1.8	0.123
Gender (boys/girls)	170/80	20/30	5/15	5/25	–
BMI	21.4 ± 3.6	24.2 ± 3.4	24.6 ± 2.1	27.1 ± 5.3	0.001
WHtR	0.41 ± 0.07	0.74 ± 0.09	0.78 ± 0.12	0.96 ± 0.12	0.001
Diet score	22.8 ± 2.3	26.7 ± 2.9	26.7 ± 2.9	31.8 ± 3.1	0.001
MVPA (%)	71.4	22.8	19.8	18.9	0.001
Blood pressure (%) Normotensive Pre-hypertensive Hypertensive	250 (100%) 0 0	30 (60%) 12 (24%) 8 (16%)	9 (45%) 10 (50%) 1 (5%)	16 (53.3% 8 (26.7%) 6 (20%)	0.003
FBG (mmol/L)	4.1 ± 0.3	4.3 ± 0.65	4.5 ± 0.94	4.8 ± 1.3	0.01
HbA1c	2.9 ± 1.6	3.1 ± 2.6	3.4 ± 2.7	3.9 ± 2.5	0.01

All values were reported as mean ± SD or median (interquartile range) or percentage. Significance was calculated by ANOVA followed by Student- Newman-Keul’s (SNK) post hoc pairwise comparison for age and metabolic parameters, or Chi-square test for residence and physical activity. ^a^, ^b^, and ^c^ refer to the difference between the study groups after pairwise comparison. Variables were considered significantly different at *P* < 0.05

NI: normal iron (iron conc.;75–175 μg/dl); ID: iron deficiency (iron conc.; < 75 μg/dl l); IDA: iron deficiency anemia (iron conc.; < 75 μg/dl l; hemoglobin < 120 g/L, and ferritin < 15 μg/L); IO: iron overload (iron conc.; ˃ 175 μg/dl) FBG: fasting blood sugar, HbA1C: glycated hemoglobin A1c; BMI, body mass index; WHtR, waist to height ratio; MVPA: Moderate-to-vigorous physical activity

### Anthropometric measurements

All students who participated in this study were subjected to determine their anthropometric measurements, such as height and weight, using a standardized procedure as previously reported [40–44]. This test used a tape measure and calibrated Salter Electronic Scales (Digital Pearson Scale; ADAM Equipment Inc., Columbia, MD, USA) to detect the height and weights of all subjects. In addition to that, previously validated universal cutoff values relating to BMI and Waist-to-height ratio (WHtR) were applied to identify both BMI and WHtR of all subjects. The validated WHtR cutoff values were based on international data from schoolchildren and previously used to recognize early cardiovascular risk in children and adolescents [40–44].

### Assessment of serum hepcidin levels and iron status

Serum hepcidin concentrations were estimated in all participants using a commercial ELISA kit (DRG Instruments, GmbH, Marburg, Germany), as previously mentioned in the literature [31]. The markers of iron status in this study were iron concentration, Hgb, serum ferritin (SF), and soluble transferrin receptor (sTfR).

Serum concentrations of iron were determined by inductively coupled plasma mass spectrometry (ICP-MS). Non-hemolyzed lipids and jaundice-free serum samples were used for the ICP-MS analysis. In this test, one millilitre of 5% HNO3 (Trace Metal Grade, Thermo Fisher, USA) solution was added to 0.5 ml serum sample to obtain a mixed solution, which was centrifuged at 12,000 rpm for 5 min to obtain the supernatant. After that, 3.5 ml 1% HNO3 solution was added to 0.5 ml supernatant, and then the mixture was centrifuged for 2 min at 2500 rpm and incubated at room temperature for 1 min to obtain the samples to be analyzed by ICP-MS (ICPMS-2030, Shimadzu, Japan). Finally, a certified commercial element standard solution (1000 mg/L) was applied to identify the accuracy and precision of the iron analysis [44–46]. Serum ferritin was measured by the Roche Tina-quant Ferritin immunoturbidimetric assay on the Hitachi 912 clinical analyzer (Roche Diagnostics)” [47]. SF “was estimated with Vitros Immunodiagnostic Products Ferritin Reagent Pack (Quest Diagnostics), sTfR was assessed with nephelometry (Quest Diagnostics). Hgb was assessed with Beckman Coulter LH 780 hematology analyzer (Quest Diagnostics)” [47]. “All three biomarkers (Hgb, SF, and sTfR) are commonly measured in studies assessing iron status during iron intake interventions” [48–50]. “Total body iron stores were also measured to evaluate tissue iron deficiencies status in many iron interventions” [2, 7, 48, 51, 52]. “It was estimated by quantifying the amount of SF and sTfR and is calculated from a validated previously reported method” [53] as follows;

{Total Body Iron Stores (mg/kg) = − [log 10 (sTfR × 1000/ SF-2.8229]/0.1207} [53]

Specifically, based on the status of iron concentrations, our participants were classified into four groups: iron deficiency group (*n* = 50; < 75 μg/dl), iron deficiency with anaemia group (*n* = 20; < 75 μg/dl and Hgb < 12.0), Normal group (*n* = 250; 75–175 μg/dl), and iron overload group (*n* = 30; high iron levels ˃ 175 μg/dl) as reported previously [54, 55].

### Assessment of total oxidative stress (OS)

Total oxidative stress (OS) was detected colourimetrically from the fresh blood samples of all the participants using a free oxygen reactive CR 3000 instrument (free oxygen reactive measurement (FORM) system, Catellani Group, Callegari S.p.A, Parma, Italy) as previously reported [56, 57]. All measurements were performed within 0.3–1 h post-blood drawing. The CR 3000 instrument includes software and photometers (505 nm) with one or three reading cells to determine primary tests on whole capillary blood. The CR 3000 kits include ready-filled cuvettes (4.8 pH buffered chromogen) that are barcoded (so that the reader automatically recognizes the test about to be performed and the *k*-factor) and capillaries for blood collection. In this test, based on Fenton’s and Haber–Weiss’s reactions, the determination of OS depends mainly on the addition of transition metals, like iron, which catalyzes the breakdown of hydroperoxides into related active free radicals that are measured consequently at a wavelength (λ; 505 nm) as shown previously [56, 57].

### Assessment of total antioxidant capacity (TAC)

Colourimetric assay kits (K274-100; BioVision, Milpitas, CA, USA) were used to determine the total antioxidant capacity (TAC) in all serum samples of all participants. The values of TAC were identified at a wavelength of (λ; 570 nm) as a function of Trolox concentration previously reported [56–58].

### Assessment of chronic inflammation

This study’s markers of chronic inflammation were α-1-acid glycoprotein (AGP) and hsCRP. As mentioned before, an in-house sandwich immune ELISA assay was applied to measure AGP in all subjects [58]. In addition, serum hsCRP concentrations were measured by the immunoturbidimetry method using a colourimetric assay kit (APTEC diagnostics NV, Belgium) on the fully automated analyzer (Beckman Synchron CX5, USA).

### Assessment of circulating RNAs

#### Extraction and purification of circulating RNA

Total RNA from all subjects’ serum was extracted using TRIzol reagent (Clontech Laboratories Inc., Mountain View, CA, USA) according to the manufacturer’s protocol. In addition to that, cDNA of miR-146a, miR-125b, and miR-122 were then synthesized using the Mir-X miRNA First-Strand Synthesis Kit (Clontech Laboratories Inc.) [58, 59]. Moreover, the integrity and quantity of total RNA were assessed using an Agilent 2100 Bioanalyzer (Agilent Technologies). As previously reported, the procedures were performed according to the manufacturer’s instructions [39, 60].

#### Real-time qPCR of microRNAs

“A ready-made solution containing the primers and probes for miR-146a, miR-155, and miR-15a (Applied Biosystems, Foster City, CA) and real-time RT–PCR was estimated using an ABI 7300 system (Applied Biosystems) [33, 39]. “Quantitative real-time polymerase chain reaction (qRT-PCR) of miR-146a, miR-125,b, and miR-122 were conducted using the Mir-X miRNA qRT-PCR SYBR Kit (Clontech Laboratories Inc.) with the Applied Biosystems 7300 Real-Time PCR System (Applied Biosystems, Foster City, CA, USA)” [33, 39, 57]. “Normalized U6 snRNA levels as an internal quantitative control and the 2 − ΔΔCt system were used to estimate the expression levels of miRNAs. Also, all reactions were run in duplicate to avoid errors and exactly determine cycle threshold mean values for each sample, including amplified miRNAs” [33, 39, 57].

### Statistical analysis

A power calculation of the selected sample size of 350 students gave an estimated power of 98% and a significance level of 0.05 with an expected frequency of 6.9%. This study used statistical software, SPSS version 17, to analyze the data obtained. The results obtained were expressed as Mean and standard deviation. Among groups, Kruskal–Wallis one-way ANOVA and post-hoc (Tukey HSD) test were used to compare the mean values of the studied variables. The relationship between various study parameters was also identified using Spearman rank correlation analysis [39]. The data *P* < 0.05 were considered significant.

## Results

Three hundred fifty school-children aged 12–18 were involved in this study. 57% of the sample was male (*n* = 200). Iron status was cross-sectionally surveyed in all participants. Normal iron status was predicted in 71.46% of the study population with average iron concentration (91.6 ± 21.3). Moreover, 28.54% of the study population (*n* = 100) had a significant status of iron disorders below and higher of normal iron ranges; they were classified into 14.26% iron deficiency without anaemia (iron conc.; < 75μg/dl l, haemoglobin ˃120 g/L, and ferritin < 15 μg/L), 5.7% showed iron deficiency with anaemia (iron conc.; < 75 μg/dl l; haemoglobin < 120 g/L, and ferritin < 15 μg/L), and 8.6% overloaded with iron concentration (iron conc.; ˃ 175 μg/dl), respectively (Table 1). In addition, adiposity and clinical parameters such as BMI, WHtR, FBS, HBA1c, diet scores, MVPA (%), and blood pressure (%) were shown to be significantly correlated with iron status, as in Table 1.

**Fig. 1:**
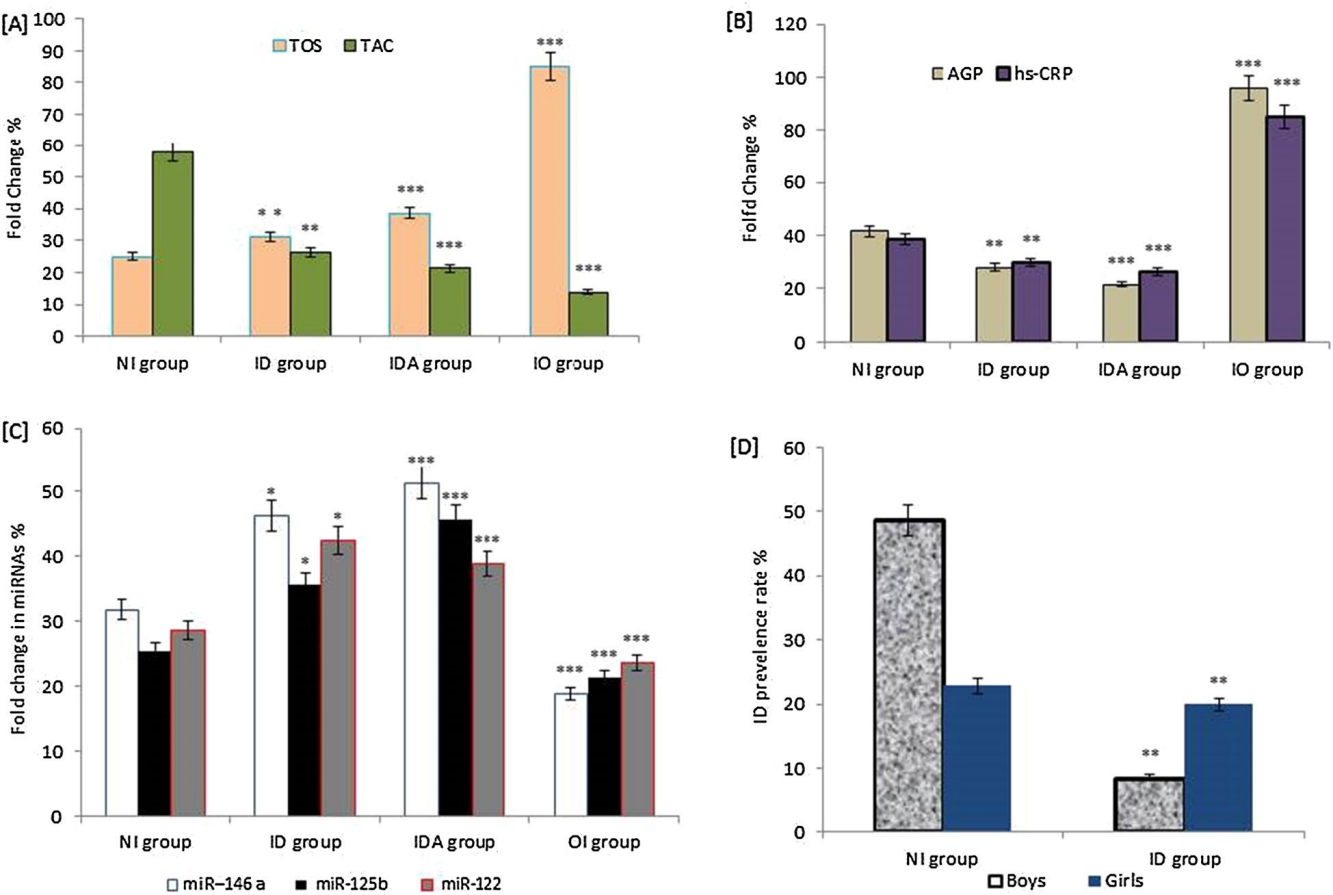
Molecular changes in oxidative stress TOS and TAC (**A**), inflammatory markers; AGP and hs-CRP (**B**), and miRNAs expression; miR-146 a, miR-125 b, miR-122 (**C**) in iron deficiency and control school children aged 12–18 years. Iron deficiency was reported according to gender (**D**). There were significant increase and decrease in the expression levels of TOS and TAC in students with ID, IDA, and iron overload status (**P = 0.01; ***P = 0.001) [**A**]. Also, AGP and hs-CRP as markers of inflammation were significantly increased in subjects with iron overload compared to those of iron disorders; ID, IDA, and controls, respectively (**P = 0.01, ***P = 0.001) (**B**). Expression of miRNAs; miR-146a and miR-125b, and miR-122 were significantly upregulated in subjects with ID, IDA, and downregulated in iron overloaded subjects compared to expression levels obtained in control subjects respectively (**P = 0.01, ***P = 0.001) (**C**). Iron deficiency was significantly higher in girls (20%; *P* = 0.01) than in boys (8.6%; *P* = 0.01) as compared to normal controls (**D**). Abbreviation: BMI: body mass index; sTfR, soluble transferrin receptor; TBIS, total boy iron store; TOS, total oxidative stress; TAC, total antioxidant capacity; hs-CRP, high sensitivity C-reactive protein; AGP, α-1-acid glycoprotein; NI: normal iron (iron conc.;75–175 μg/dl); ID: iron deficiency (iron conc.; < 75 μg/dl l); IDA: iron deficiency anemia (iron conc.; < 75 μg/dl l; hemoglobin < 120 g/L, and ferritin < 15 μg/L); IO: iron overload (iron conc.; ˃175 μg/dl). Variables were considered significantly different at *P* < 0.05

In the present study, the overall proportion of iron depletion was significantly higher in girls (20.0%) than in boys (8.6%), as shown in Fig. 1D.

Table 2 shows the association of iron parameters with iron status among school children. Lower iron conc., ferritin, transferrin saturation, TBIS, and hepcidin serum levels in association with higher levels of sTfR (nmol/L) were significantly (*P* = 0.001) reported in subjects with iron deficiency (ID) compared to those of normal iron status. In subjects with iron deficiency with anaemia (IDA), a significant decrease in all iron parameters, iron conc., ferritin, transferrin saturation, TBIS, hepcidin serum levels, sTfR, along with lower Hgb % were estimated in the IDA group compared to those of control and ID groups respectively (*P* = 0.001). However, in overloaded subjects, significant (*P* = 0.001) increase in iron concentration, serum hepcidin, and decrease in ferritin, transferrin saturation, TBIS, and sTfR, in association with normal Hgb levels were recorded in all subjects compared to other iron status groups (Table 2).

**Table 2:** Measurements of hemoglobin, iron parameters, and levels of hepcidin in schoolchildren with different iron scores

Variables	Iron status				*P*-value
	N I group (*n* = 250)	ID group (*n* = 50)	IDA group (*n* = 20)	IO group (*n* = 30)	
Hepcidin (ng/ml)	68.6 ± 8.1	18.6 ± 6.1	14.6 ± 5.2	56.8 ± 3.7	0.001
Ferritin (ng/ml)	48.6 ± 15.7	32.6 ± 12.3	11.8 ± 6.9	9.5 ± 2.4	0.001
sTfR (nmol/L)	35.4 ± 7.4	52.9 ± 11.59	8.9 ± 3.1	7.1 ± 3.8	0.001
Transferrin saturation (%)	21.7 ± 6.5	13.9 ± 5.4	5.7 ± 3.8	19.5 ± 2.3	0.001
Iron (μg/dl)	91.6 ± 21.3	41.8 ± 3.4	21.5 ± 3.4	120.4 ± 31.4	0.001
TBIS	3.88 ± 1.1	2.6 ± 1.3	1.9 ± 0.85	7.6 ± 2.3	0.001
Hgb (g/dl)	13.2 ± 1.7	12.6 ± 2.1	8.6 ± 1.9	12.1 ± 2.7	0.001

All values were reported as mean ± SD or median (interquartile range) or percentage. Significance was calculated by ANOVA followed by Student- Newman-Keul’s (SNK) post hoc pairwise comparison for age and metabolic parameters, or Chi-square test for residence and physical activity. Variables were considered significantly different at *P* < 0.05

NI: normal iron (iron conc.; 75–175 μg/dl); ID: iron deficiency (iron conc.; < 75 μg/dl l); IDA: iron deficiency anemia (iron conc.; < 75 μg/dl l; hemoglobin < 120 g/L, and ferritin < 15 μg/L); IO: iron overload (iron conc.; ˃ 175 μg/dl); sTfR, soluble transferrin receptor; TBIS, total boy iron store; Hgb, hemoglobin

In this study, inflammation and oxidative stress associated with iron deficiency or overload were significantly estimated in all participants. Oxidative stress parameters, TOS, and TAC were shown to be significantly correlated with iron status. The data showed a significant increase in the levels of TOS and a decrease in TAC among students with iron disorders, ID, and IDA, especially in iron-overloaded subjects compared to those of normal iron, as in Fig. 1A. Also, inflammatory markers, AGP and hs-CRP, were estimated in all subjects with different iron statuses. AGP and hs-CRP as markers of inflammation were significantly increased in iron-overloaded subjects compared to normal controls, ID, and IDA, respectively (*P* = 0.01, *P* = 0.001), as shown in (Fig. 1B).

In this study, the role of miRNA in modulating iron homeostasis was significantly estimated in all participants. The data obtained showed that the expression of miRNAs; miR-146a and miR-125b, and miR-122 was significantly up-regulated (increased) in subjects with ID and IDA and downregulated (decreased) in control subjects with iron-overloaded, respectively (*P* = 0.01, *P* = 0.001) as shown in Fig. 1C.

Both hepcidin and miRNA expression levels were significantly correlated with parameters of iron status, inflammation, and oxidative stress. They were positively associated with plasma concentrations of Hgb, AGP, hs-CRP, TAC, iron conc., ferritin, transferrin saturation (%), and negatively with TBIS, sTfR, and TOS as biomarkers of metabolic iron disorders in ID, IDA, and overloaded subjects, respectively (Table 3). In addition, miRNAs miR-146a and, miR-125b, and miR-122 correlated negatively with BMI, WHtR, diabetic control variable (HbA1c), and no significance was reported with age and gender of the studied subjects as shown in Table 3.

**Table 3:** Correlations between serum hepcidin and other circulating miRNAs parameters

Variables	Serum hepcidin		miRNAs expression					
			miR-146a		miR-125b		miR-122	
	*r*	*P*	*r*	*P*	*r*	*P*	*r*	*P*
Age	− 0.125	0.11	− 0.29	0.31	− 0.68	0.13	− 0.29	0.12
Gender	− 0.142	0.18	− 0.15	0.17	− 0.37	0.14	− 0.31	0.147
BMI	− 0.35	0.01	− 0.42	0.001	− 0.56	0.001	− 0.36	0.001
WHtR	− 0.21	0.01	− 0.35	0.001	− 0.48	0.001	− 0.72	0.001
HbA1c	− 0.18	0.01	− 0.21	0.001	− 0.38	0.001	− 0.42	0.001
Ferritin (ng/ml)	0.62	0.001	0.75	0.001	0.89	0.001	0.96	0.001
sTfR (nmol/L)	− 0.85	0.001	− 0.65	0.001	− 0.56	0.001	− 0.76	0.001
Transferrin saturation (%)	0.45	0.001	0.49	0.001	0.58	0.001	0.78	0.001
Iron (μg/dl)	0.39	0.01	0.52	0.01	0.63	0.01	0.82	0.01
TBIS	− 0.58	0.001	− 0.67	0.001	− 0.75	0.001	− 0.95	0.001
TOP	− 0.38	0.01	− 0.54	0.01	− 0.62	0.01	0.54	0.01
TAC	0.25	0.001	0.36	0.001	0.45	0.001	0.68	0.001
AGP	0.45	0.01	0.67	0.01	0.78	0.01	0.85	0.01
hs-CRP	0.189	0.01	0.165	0.01	0.198	0.01	0.178	0.01
Hgb	0.25	0.01	0.37	0.01	0.39	0.01	0.058	0.01

Data are r (spearman)

*BMI* body mass index, *sTfR* soluble transferrin receptor, *TBIS* total boy iron store, *TOS* total oxidative stress, *TAC* total antioxidant capacity, *hs-CRP* high sensitivity C-reactive protein, *AGP* α-1-acid glycoprotein. Variables were considered significantly different at *P* < 0.05

Adjusted linear regression analyses demonstrated that miRNAs; miR-146a and miR-125b, and miR-122 were positively associated with hepcidin, ferritin, iron, transferrin saturation, TAC, and haemoglobin and negatively associated with sTfR, TIBs, AGP,hs-CRP, and TOS (Table 4).

**Table 4:** Adjusted^a^ simple linear regression for miRNAs expression in iron disorders

Variables	miRNAs expression					
	miR-146a		miR-125b		miR-122	
	β (95% CI)	*P*	β (95% CI)	*P*	β (95% CI)	*P*
Hepcidin	0.18 (0.10–0.28)	0.01	0.36 (0.22–0.48)	0.001	0.42 (0.31–0.58)	0.001
Hgb	0.235 (0.185–0.250)	0.01	0.332 (0.296–0.398)	0.001	0.242 (0.201–0.315)	0.001
Ferritin (ng/ml)	0.026 (0.015–0.029	0.01	0.032 (0.026–0.038	0.001	0.042 (0.032–0.056	0.001
sTfR (nmol/L)	− 0.038 (0.034–0.052	0.001	− 0.028 (0.021–0.039	0.001	− 0.049 (0.036–0.065	0.001
Transferrin saturation (%)	0.058 (0.046–0.078)	0.001	0.068 (0.056–0.086)	0.001	0.084 (0.068 -0.098)	0.001
Iron (μg/dl)	0.18 (0.012–0.028)	0.001	0.021 (0.016–0.029)	0.001	0.031 (0.025–0.048)	0.001
TBIS	− 0.008 (0.002–0.012)	0.001	− 0.013 (0.009–0.018)	0.001	− 0.025 (0.018–0.031)	0.001
AGP	− 0.325 (0.299–0.360)	0.001	− 0.245 (0.215–0.296)	0.001	− 0.125 (0.112–0.180)	0.001
hs-CRP	− 0.428 (0.391–0.516)	0.001	− 0.360 (0.256–0.415)	0.001	− 0.186 (0.165–0.230)	0.001
TOS	− 0.125 (0.98–0.210)	0.001	− 0.198 (0.156–0.261)	0.001	− 0.210 (0.195–0.315)	0.001
TAC	0.38 (0.26–0.52)	0.001	0.48 (0.36–0.75)	0.001	0.31 (0.22–0.46)	0.001

^a^Adjusted for age, gender, and body mass index

*Hgb* hemoglobin, *sTfR* soluble transferrin receptor, *TBIS* total boy iron store, *TOS* total oxidative stress, *TAC* total antioxidant capacity, *hs-CRP* high sensitivity C-reactive protein, *AGP* α-1-acid glycoprotein, *CI* confidence interval

## Discussion

Iron status was cross-sectionally surveyed in 350 school children aged 12–18 in this study. Normal iron status was predicted in 71.46% of the study population, and 28.54% of the study population had significant changes in iron status; they were classified into iron deficiency without anaemia (ID) (14.26%), showed iron deficiency with anaemia (IDA) (5.7%), and overloaded with iron concentration (8.6%) respectively.

Previous research studies reported that ID and ADA are common nutritional disorders that place children at significant health risks, such as general weakness in body growth, a compromise in the ability of the immune system, increasing morbidity, delay in psychomotor or mental development, and poor cognitive performance which lead to lower schooling achievements [4–6, 59]. Across various African and Asian settings of developing and non-developing countries, 40% of children suffer from an iron deficiency with anaemia [4].

In this study, iron deficiency was significantly higher among girls (20%) than boys (8.6%). The data obtained are in line with previous findings, which reported a higher decline in iron status among European adolescents, especially girls, who reported higher ID rates (43%) compared to boys (16%) [60–62].

In adolescents, higher ID rates were reported in girls and boys, especially during the growth periods, as they need adequate amounts of iron for increasing muscular growth [63]. In girls, in addition to the fast growth, the onset of menstruation also leads to iron losses [64]. Several biochemical iron indicators, mainly iron conc., ferritin, transferrin saturation, and TBIS, were evaluated as laboratory diagnostic indicators ID in younger populations [65, 66].

In the current study, iron parameters iron conc., Hgb, ferritin, transferrin saturation, TBIS, and sTfR were significantly associated with iron status among all school adolescents. Lower iron conc., serum ferritin (SF), transferrin saturation, and TBIS, with higher levels of sTfR (nmol/L), were significantly (*P* = 0.001) reported in subjects with iron deficiency (ID). However, significant (*P* = 0.001) depletion in all iron indicators, including lower levels of Hgb %, was reported in subjects with IDA. In overloaded subjects, a significant (*P* = 0.001) increase in iron concentration and a decrease in ferritin, transferrin saturation, TBIS, and sTfR, in association with normal Hgb levels, were recorded in all subjects compared to other iron status groups. The data matched other findings that reported that Hgb, iron concentration, SF, and soluble transferrin receptor (sTfR) were the most commonly utilized markers of iron deficiency in clinical practice. The levels of these indicators efficiently reflect intracellular iron stores [2, 7, 8]. Also, SF and sTfR underwent a characteristic sequence of changes following a decrease in body iron stores from normal iron-replete levels to those found in ID [9–12].

Iron deficiency or overload leads to abnormal iron metabolism and severe multiple-organ dysfunctions[67, 68]. Abnormal iron metabolism consequences such as cardiomyopathy may lead to higher morbidity and mortality in iron-overloaded patients [69–72]. Thus, iron levels must be maintained within physiological limits among children and adolescents to avoid pathological manifestations [69–72]. Also, the most common causes of abnormal iron metabolism were inflammation and a biological increase in oxidative stress parameters in tissues and organs. Excess iron promotes the generation of reactive oxygen species (ROS) and increases oxidative stress, consequently damaging parenchymal tissues [69].

In our study, high sensitive C-reactive protein (hs-CRP), which is a measure of acute inflammation, and a1-acid glycoprotein (AGP), which is a measure of chronic inflammation, along with total oxidative stress (TOS) and total antioxidant capacity (TAC) were evaluated in all subjects. Significant increases in AGP, hs-CRP, TOS levels, and a decrease in TAC activity were reported in school adolescents with different iron disorders: ID, IDA, and iron overload cases, respectively (*P* = 0.01, *P* = 0.001). In particular, acute-phase response (APR) inflammation can directly affect most iron indicators’ concentrations [73, 74]. Both SF and sTfR concentrations appear to significantly increase in individuals with general inflammation [75, 76]. Also, the elucidation of total body iron stores (TBIs) is controlled by both SF and sTfR concentrations, whereas TBIs are the net result of a combination of these indicators [77].

Previous studies recommend conducting surveys in low-inflammation scores or measuring inflammatory indicators in case of using SF concentrations to assess deficiency in iron status. They recommend CRP/hs-CRP and AGP, which are measures of acute and chronic inflammation [78–80]. The biological mechanisms that underpin the effect of inflammation and related oxidative stress consequences on iron-status indicators among children and adolescents still need to be fully elucidated. Most studies suggested that inflammation may affect iron status by decreasing food intake and minimizing intestinal absorption [74, 81], leading to bidirectional interactions between inflammation and malnutrition and directly affecting immune function and the APR [74].

Thus, to understand the molecular mechanism of the effect of inflammation and related oxidative stress consequences on iron status, new approaches based on the use of biomarkers of hepcidin and miRNAs to interpret iron indicators are emerging.

Hepcidin levels reflected the integration of multiple key signals involved in iron regulation. It directly controls iron absorption and bioavailability in circulation. So, it is considered a useful clinical tool for managing iron disorders [81–83]. Hepcidin as a liver-derived peptide hormone was estimated and correlated with iron status in all subjects. Lower levels of serum hepcidin were significantly (*P* = 0.001) reported in subjects with iron deficiency (ID) and iron deficiency with anaemia (IDA), and highly expressed levels were recorded in iron-overloaded subjects, respectively. The data showed that hepcidin correlated positively with Hgb, AGP, hs-CRP, TAC, iron conc., ferritin, transferrin saturation (%), and negatively with TBIS, sTfR, and TOS as biomarkers of metabolic iron disorders in ID, IDA, and overloaded subjects, respectively. In addition, hepcidin also correlated negatively with BMI, WHtR, and diabetic control variables (HbA1c). Hepcidin, a newly discovered harmful iron regulatory hormone, can explain iron deficiency and anaemia among cases of chronic inflammation and infections [22].

In inflamed cellular tissues, the production of proinflammatory cytokine interleukin-6 within inflamed cells significantly activates mainly the liver to release hepcidin [23–26]. The expressed liver hepcidin suppresses intestinal iron absorption and storage iron release [23–26]. In addition to that, hepcidin interacts with the iron-exporter ferroportin to control the amount of iron available in circulation [23–26].

During iron supplementation, the success of host iron absorption was master regulated by cellular hepcidin, as it controls the incorporation of iron into erythrocytes [27]. Moreover, a disorder in the hepcidin/ferroportin regulatory system was shown to be associated with an imbalance of systemic iron levels and is consequently related to different severe iron disorders, such as iron deficiency anaemia [28], iron overload disorders [29], and thalassemias [30].

Although in vitro analysis studied the role of hepcidin in iron metabolism, more data were needed from human studies, especially those of healthy subjects [31]. Thus, more research studies try to investigate the role of miRNAs in iron homeostasis and possible cases of anaemia in healthy subjects. miRNAs were suggested to regulate up to 30% of the human protein-coding genes as determined by computational analysis [33]; however, only a small number of target genes have been experimentally confirmed [34].

In this current study, the role of miRNA in modulating iron homeostasis was significantly estimated in all participants. The data obtained showed that the expression of miRNAs, such as miR-146a, miR-125b, and miR-122, was significantly upregulated (increased) in subjects with different iron statuses: ID, IDA, and differentially decreased in overload subjects compared to expression levels obtained in control subjects (*P* = 0.01, *P* = 0.001).

Previous studies suggested that miRNA may also have a regulatory effect on human iron homeostasis. The role of miRNA in modulating iron homeostasis is proposed to be at least a part of a heme-dependent process [35, 36]. MiRNAs potentially regulate systemic and cellular iron homeostasis at multiple points by influencing iron absorption, transport, storage, and utilization [37]. Previously, it was suggested that miRNA may also be critical regulators in many facets of human iron homeostasis, at least through a heme-dependent process [84, 85].

Many studies showed that miR-122 is an abundant, liver-specific miRNA miR-122 plays an essential role in regulating hepatic functions [19, 86, 87]. The upregulation of mir-122 in liver cells was shown to regulate iron disorders via controlling hepcidin expression. It was proposed that miR-122 expression significantly affects mRNA transcribed by genes that control systemic iron levels, such as hemochromatosis (Hfe), hemojuvelin (Hjv), bone morphogenetic protein receptor type 1A (Bmpr1a), and Hamp. These genes, especially Hfe and Hjv, are responsible for encoding activators of hepcidin expression, which are repressed by the expression miR-122 in lower systemic iron levels [28, 39, 88, 89]. However, in cases with higher iron status, downregulation of miR-12 significantly activates genes that encode activators of hepcidin expression, Hfe, and Hjv. Subsequently, it increases hepcidin levels of iron-overloaded subjects to hamper or regulate excess iron accumulation in body cells, especially liver cells. Thus, miR-122 was suggested to have a direct mechanistic link with regulating systemic iron metabolism [28, 39, 88–90].

Recently, “it was found that iron concentrations significantly associated with synergistic in upregulating Reactive Oxygen Species (ROS) abundance which biogenetically regulate the expression of miRNA-125b and miRNA-146a [91], suggesting the role of iron in modulating the biogenesis of miRNAs; miRNA-125b and miRNA-146a by oxidative stress pathway in our study. Also, another study proposed that exposure to iron changed microRNA expression in opposite directions [92]. This urge that iron may be involved in the Drosha/DGCR8/heme-mediated processing of microRNAs [93].

In adolescents with iron disorders, positive correlations were found between the expression of miRNAs; miR-146a and miR-125b, and miR-122 and hepcidin, ferritin, iron, transferrin saturation, TAC, and haemoglobin; conversely, negative correlations were reported with sTfR, TIBs, AGP,hs-CRP, and TOS.

Recently, miRNA expression has been shown to regulate systemic and cellular iron homeostasis at multiple points by influencing physiological processes like iron absorption, transport, storage, and utilization. The expressed miRNAs might increase TfR expression and decrease the level of ferritin protein abundance and haemoglobin content in the cell [94–97].

Also, the miRNAs expression may control cellular iron homeostasis through regulation of iron storage via induction of the expression of both forms of the iron storage protein, heavy- or heart ferritin (FtH) and light- or liver ferritin (FtL) [40, 98–101].

Finally, these exciting findings highlight the potential role of miRNAs; miR-146a, miR-125b, and miR-122, to provide an additional means of controlling or regulating cellular iron uptake or metabolism either via the oxidative stress pathway [91, 92] or the regulation of genes encoding Hfe and Hjv activators, which are responsible for encoding activators of hepcidin expression [28, 39, 88–90].

## Conclusion

Iron disorders were cross-sectionally predicted in 28.54% of the study population; they were classified into 14.26% with ID, 5.7% with IDA, and 8.6% with iron overload. The overall proportion of iron depletion was significantly higher in girls (20.0%) than in boys (8.6%). Serum hepcidin was shown to be correlated with the magnitude disorder in the iron status measured by iron concentration and other iron parameters among subjects with ID and IDA, respectively. Thus, it was postulated as an iron regulator and might be a useful marker in diagnosing or treating iron deficiency, especially in subjects with ID or IDA. In addition, molecular identification of miRNAs; miR-146a, miR-125b, and miR-122 were shown to control the cellular iron uptake or metabolism via the oxidative stress pathway or regulation of hepcidin expression via activating genes encoding Hfe and Hjv activators, which promote iron regulation. Thus, these molecular markers, along with standard markers, could provide an additional means of controlling or regulating cellular iron and could be associated as valuable markers in diagnosing and treating cases with different iron deficiencies.

## Data Availability

The datasets supporting the conclusions of this article are included within the article.
